# Activation of IGFBP4 via unconventional mechanism of miRNA attenuates metastasis of intrahepatic cholangiocarcinoma

**DOI:** 10.1007/s12072-023-10552-7

**Published:** 2023-06-22

**Authors:** Liye Tao, Yali Wang, Zefeng Shen, Jingwei Cai, Junhao Zheng, Shunjie Xia, Zhongjie Lin, Zhe Wan, Haiou Qi, Renan Jin, Ling Wang, Junjie Xu, Xiao Liang

**Affiliations:** 1 Key Laboratory of Laparoscopic Technology of Zhejiang Province, Department of General Surgery, Sir Run-Run Shaw Hospital, Zhejiang University School of Medicine, Hangzhou, 310016 China; 2 Zhejiang Research and Development Engineering Laboratory of Minimally Invasive Technology and Equipment, Zhejiang Minimal Invasive Diagnosis and Treatment Technology Research Center of Severe Hepatobiliary Disease, Hangzhou, 310016 China; 3 Zhejiang University Cancer Center, Hangzhou, 310058 China; 4 Liangzhu Laboratory, Zhejiang University Medical Center, Hangzhou, 311121 China; 5 Nursing Department and Nurse of Operating Room, Sir Run Shaw Hospital, Zhejiang University School of Medicine, Hangzhou, China; 6 School of Automation, Hangzhou Dianzi University, Hangzhou, China; 7 Key Laboratory of Medical Information and 3D Bioprinting of Zhejiang Province, Hangzhou, China

**Keywords:** Intrahepatic cholangiocarcinoma, Metastasis, miR-122-5p, IGFBP4

## Abstract

**Background:**

Intrahepatic cholangiocarcinoma (ICC) is the second most common primary liver malignancy. Although its incidence is lower than that of hepatocellular carcinoma (HCC), ICC has a worse prognosis, and it is more prone to recur and metastasize, resulting in a far greater level of malignancy.

**Methods:**

Bioinformatics analysis and qRT-PCR were applied to assess the level of miR-122-5p and IGFBP4. Western blot, transwell assays, wound-healing assays, real-time cellular invasion monitoring, in vivo study were applied to explore the function of miR-122-5p and IGFBP4. Dual luciferase reporter assays and chromatin isolation by RNA purification (ChiRP) were applied to explore the regulation of IGFBP4 by miR-122-5p.

**Results:**

Using The Cancer Genome Atlas (TCGA) data set, Sir Run Run Shaw hospital data set and bioinformatics analyses, we identified miR-122-5p as a potential tumor suppressor in ICC and validated its suppressive effect in metastasis and invasion of ICC. Transcriptome sequencing, rescue and complement experiments were used to identify insulin-like growth factor binding protein 4 (IGFBP4) as a target of miR-122-5p. The mechanism by which miR-122-5p regulates IGFBP4 was clarified by chromatin separation RNA purification technology, and dual-luciferase reporter assays. We discovered a rare novel mechanism by which miR-122-5p promotes IGFBP4 mRNA transcription by binding to its promoter region. Furthermore, in mouse orthotopic metastasis model, miR-122-5p inhibited the invasion of ICC.

**Conclusion:**

In summary, our study revealed a novel mechanism of miR-122-5p and function of the miR-122-5p/IGFBP4 axis in the metastasis of ICC. We also highlighted the clinical value of miR-122-5p and IGFBP4 in inhibiting ICC invasion and metastasis.

**Supplementary Information:**

The online version contains supplementary material available at 10.1007/s12072-023-10552-7.

## Introduction

Intrahepatic cholangiocarcinoma (ICC) is the second most common primary malignant tumor in the liver, accounting for approximately 15% of all primary liver cancers [1], and its incidence has grown tenfold in the last three decades [2]. The incidence of ICC varies widely around the world, ranging from approximately 0.35 cases/100,000 to 2 cases/100,000 in high-income Western countries. In some regions, such as Thailand and China, the incidence of ICC is significantly higher (approximately 40-fold) than that in developed western countries [3, 4]. Currently known risk factors for ICC include choledochal cysts, primary sclerosing cholangitis, biliary cirrhosis, cholelithiasis, thoracoscopy, Caroli disease, hepatobiliary flukes, and cirrhosis [5–8]. Although various treatments for ICC exist, including surgery, local therapy, radiotherapy, chemotherapy, and immunotherapy, the prognosis of ICC remains poor, mainly because ICC is extremely prone to metastasis and recurrence. ICC cells will gradually acquire an invasive phenotype and metastasize, likely through epithelial–mesenchymal transition (EMT), thereby acquiring a mesenchymal phenotype. This transformation increases the ability of ICC cells to migrate and invade and eventually colonize distant sites [9, 10]. At present, there is no effective treatment that significantly inhibits the metastasis and recurrence of ICC.miRNAs are short fragments of non-coding RNAs of 17–25 nucleotides in length. miRNA synthesis consists of multiple steps, including transcription, nuclear seeding, transport to the cytoplasm, and intracytoplasmic cleavage [11]. The main mechanism of miRNAs is inhibition of the expression of target genes through binding to the 3′UTR of the target gene mRNA via its seed region [12]. In addition to the aforementioned conventional mechanisms, recent studies have identified seven rare and unconventional mechanisms as follows: pri-miRNAs encode polypeptides, miRNAs bind to non-Ago2 proteins, miRNAs activate Toll-like receptors, miRNAs upregulate protein expression, miRNAs regulate mitochondrial gene transcription, miRNAs directly activate mRNA transcription, and miRNAs regulate nuclear non-coding RNAs [13]. Regarding the miRNAs that directly activate mRNA transcription, Place et al. found that miR-373 could target the promoter sequence of E-cadherin and induce gene expression [14]. Matsui et al. reported that miR-589 could bind promoter RNA and activate COX-2 transcription [15]. Bai et al. discovered that miR-195-5p could activate the transcription of Foxo3 by targeting its promoter region [16].

Insulin-like growth factor (IGF) binding proteins (IGFBPs) comprise a family of six proteins, namely IGFBP1–IGFBP6. Originally, they were considered the regulatory proteins of IGF-I and IGF-II in blood, and the binding of IGF to IGFBP results in increased stability [17]. Later, it was gradually discovered that IGFBPs regulate both the IGF1R signal transduction pathway by affecting the half-life of IGF and the function of cells through an IGF1R-independent pathway [18]. IGFBP4 is the smallest protein in the IGFBP family [19]. Previous studies reported that in lung cancer, the promoter region of IGFBP4 is hypermethylated, and the expression of IGFBP4 is negatively correlated with that of Ki-67 [20]. In addition, the binding of IGF-I to IGFBP4 can increase the stability of IGFBP4 protein and increase its susceptibility to degradation by proteolytic enzymes [21]. In gastric cancer, IGFBP4 is positively correlated with well-differentiated cells. When cells undergo spontaneous differentiation, IGFBP4 content gradually increases [22, 23]. In hepatocellular carcinoma (HCC), IGFBP4 was reported as a tumor suppressor that was negatively associated with cell invasiveness [24]. In addition to HCC, IGFBP4 can impair the invasiveness of colorectal cancer and lung cancer cells [25, 26]. Overall, the existing literature suggests that IGFBP4 can inhibit tumor progression [27].

In this study, we aimed to identify miRNAs involved in the migration and invasion of ICC by analyzing differentially expressed miRNAs from paired ICC tumor and adjacent normal tissue samples. We found a set of miRNAs, including miR-122-5p, which were altered in ICC tumors compared with their expression in normal tissue. Overexpression of miR-122-5p inhibited the migration and invasion of ICC in vitro and in vivo. We further found that IGFBP4 was the target protein of miR-122-5p, and miR-122-5p could directly bind to the promoter region of IGFBP4. Our work demonstrated a rare and unconventional mechanism of miR-122-5p and implicated the therapeutic value of miR-122-5p and IGFBP4 in ICC.

## Materials and methods

### Cell culture and transfections

Four cell lines of cholangiocarcinoma (CCLP1, HuCCT1, RBE, 9810), the bile duct epithelial cell line (HIBEC) cells were obtained from the American Type Culture Collection and were maintained in Dulbecco modified Eagle medium containing 10% (v/v) fetal bovine serum at 37 degree in 5% CO_2_ condition. All cell lines were routinely tested to be negative for mycoplasma contamination. Transfection of siRNA and plasmids was performed using Lipofectamine 3000 Reagents (Thermo Fisher Scientific, Waltham, MA) following the manufacturer’s instructions. The sequences of siRNAs against specific targets were listed in Table S1.

### RNA extraction and qRT-PCR assay

Cells were transiently transfected with indicated plasmids or siRNA for 24–48 h, and the total RNA was then isolated using TRIzol Reagent (15596–018; Invitrogen, Carlsbad, CA). The concentration of RNA was measured by NanoDrop 2000. Total RNA was then transcribed into complementary DNA using Hifair II 1st Strand cDNA Synthesis Kit (11121ES60, Yeasen Biotechnology) according to the manufacturer’s protocol. The quantification of complementary DNA was performed using SYBR Green Master Mix (11201ES08-5, Yeasen Biotechnology) on LightCycler 480 (Roche, Basel, Switzerland). The housekeeping gene, glyceraldehyde-3-phosphate dehydrogenase, was used as a loading control. The sequences of primers against specific targets were listed in Table S2.

### Cell proliferation assay

Transiently transfected cells were seeded in a 96-well (0.5–1 × 10^4^/well) with three replicates. Cell viability was then assessed by the Cell Counting Kit-8 (CCK-8) kit (Yeasen).

### Wound healing assay

To evaluate the migration ability of cells, the wound-healing assay was performed. Transiently transfected cells were seeded in both chamber (1 ~ 2 × 10^5^/well) of wound healing assay chamber in 6-well plate with three replicates. After the cells adhered, the chamber was removed, and the scratches between the two chambers were photographed every 12 h to observe the crawling of the cells.

### Transwell assay

To evaluate the migration and invasion ability of cells, the transwell assay was performed. For the migration assays, transiently transfected cells were resuspended in serum-free medium and seeded in upper chamber (2–6 × 10^4^/well) of 24-well transwell inserts (Transwell^™^ Permeable Polyester Membrane Inserts from Corning Inc.) with 8 μm-pore size membranes. For invasion assays, transiently transfected cells were resuspended in serum-free medium and seeded in Matrigel-coated upper chamber (2–6 × 10^4^/well) of 24-well transwell inserts. FBS-supplemented medium was added to the bottom of each transwell. After incubated at 37 °C with 5% CO_2_ for 24 or 48 h, chambers were fixed with ice cold 100% methanol for 20 min, stained with 0.1% Crystal Violet for 20 min at room temperature, and then every well was photographed at 20×. Experiments were performed in triplicate. Representative images are presented.

### Bioinformatic analysis

The Weighted gene correlation network analysis (WGCNA) was used to build the circulating miRNA co-expression network according to the procedure of the WGCNA package in R language [46]. Firstly, the gene expression similarity matrix was constructed by calculating the absolute value of the Pearson’s correlation coefficient between gene pairs. Subsequently, the gene expression similarity matrix was converted into and an adjacency matrix by using a power adjacency function, which encodes the strength of the connection between node pairs. According to the scale-free topological algorithm, the adjacency matrix met the scale-free topology criterion when *R*2 value was approximated to 0.80.

To identify hub gene, modules associated with the clinical trait of interest, were further considered for the intranodal analysis. The significance cut-off for the module selection was *p* = 0.05. Within the module, the gene significance (GS) and module membership (MM) were considered [47]. For details in the procedure please refer to the works of Langfelder et al. and Zhang et al [46, 48].

### Cell cycle analysis

Following the designated treatments, cells were trypsinized with Trypsin/EDTA (0.25%) (Gibco by Life Technologies, Grand Island, NY, USA) and then washed with PBS and fixed in ice-cold 75% ethanol overnight at − 20 °C. Fixed cells were washed and dissolved in RNAse and permeabilized with 0.1% Triton X-100 and subsequently stained with propidium iodide (PI) incubated at 37 °C for at least 30 min. The DNA content of the cells was determined using a BDLSRII flow cytometer (BD Biosciences, Franklin Lakes, NJ, USA).

### Western blot analysis

Cells were lysed in lysis buffer and proteins (30 µg) were separated on 10–12% SDS/PAGE gel and then transferred onto PVDF membranes (Millipore, Billerica, MA). After blocking membranes, they were incubated with appropriate dilutions (1:2000 for internal control antibodies and 1:1000 for others) of specific primary antibodies against β-tubulin, GAPDH, β-Catenin, ZEB1, ZO-1, N-cadherin, E-cadherin, Vimentin, Slug, Snail, Claudin-1 (from CST). The blots were incubated with HRP-conjugated secondary antibodies and visualized using the ECL system (Thermo Fisher Scientific, Rochester, NY).

### RNA fluorescence in situ hybridization (FISH)

We used the FISH kit from GenePharma (Shanghai, China) according to the manufacturer's protocol. The probe for miR-122-5p was 5′- CAAACACCATTGTCACACTCCA -3′.

### Luciferase reporter assay

The promotor of IGFBP4 containing the potential miR-122-5p binding site was synthesized by TSINGKE Biological Technology (Beijing, China). The sequence was cloned into pGL3-basic (Promega, USA). The promotor of IGFBP4 was inserted at the head of the firefly luciferase gene and the Renilla luciferase gene was used as an internal control. Cells transfected with IGFBP4 promotor plasmid and Renilla luciferase plasmid were then transfected with miR-122-5p and negative control after 7 days waiting for the plasmid enter the nucleus to form a compact form. The Promega Dual-Luciferase Reporter assay system (Promega, USA) was used to measure the activities of firefly and Renilla luciferase. The promotor sequence of IGFBP4 was listed in Table S3.

### Chromatin isolation by RNA purification (ChiRP)

We used the ChiRP kit from Ruibo (Guangzhou, China) according to the manufacturer's protocol. The probe for IGFBP4 promotor region was hsa-miR-122-5p mimic tagged with biotin. (5′- UGGAGUGUGACAAUGGUGUUUG-biotin -3′).

### Immunohistochemistry

Tumor tissues from Sir Run-Run Shaw Hospital, Zhejiang University were used to examine IGFBP4 expression. Patients’ information is listed in the supplementary data (Fig. S3) Immunohistochemistry was performed according to the previous report. Sections (3-μm-thick) were cut from routinely processed formalin-fixed, paraffin-embedded tissue blocks and subjected to immunohistochemistry staining with specific primary antibodies against IGFBP4. The slides were incubated with the primary antibody at 4 °C overnight. After washing with PBS, slides were processed using the GTvision immunohistochemistry kit according to the manufacturer’s protocol.

### Multiplex fluorescent immunohistochemistry (mIHC) staining

Multiplex staining of CK19, IGFBP4 and Slug co-expression on cancer tissues was performed using 4-color kit (WiSee Biotechnology), according to manufacturer's instruction. Three primary antibodies are anti-Cytokeratin 19 (Cat: ab52625, diluted 1:400, Abcam), anti-IGFBP4 (Cat: 18500-1-AP, diluted 1:500, proteintech) and anti-SNAIL2 (Cat: 12129-1-AP, diluted 1:400, proteintech). After applied different primary antibodies (anti-Cytokeratin 19, anti-IGFBP4 and anti-SNAIL2, sequentially), the secondary antibody (HRP conjugated) was added and incubated, followed by tyramide signal amplification (Cat: M-D110051, WiSee Biotechnology). After all antigens being labeled with different antibodies, DAPI (Cat: D1306, ThermoFisher) was used for nuclei staining. Pannoramic MIDI imaging system (3D HISTECH) was then used for scanning the stained slides. The number of target cells were analyzed by HALO software (Indica Labs).

### In vivo studies

For the patient-derived xenograft (PDX), the liver tumor specimen of a 59-year-old male patient who had undergone hepatectomy at Sir Run-Run Shaw Hospital, Zhejiang University (Hangzhou, China) was obtained. Informed consent was obtained from the patient, and the study protocol was approved by an institutional review committee of Sir Run-Run Shaw Hospital, Zhejiang University. The specimen was directly implanted into the subcutaneous space of a NOD/SCID mouse (Shanghai SLAC laboratory Animal Co., Ltd.). After two months, the NOD/SCID mouse was euthanized and the engrafted tumor was implanted into 20 nude mice in liver. When the tumors reached a length of ~ 3 mm, mice were randomized into two groups and assigned to receive lenti-vector, lenti-pre-122 injection every week. Tumor volume was calculated using digital caliper measurements. Mice were euthanized for analysis on week 4 and week 8. Tumors were harvested and frozen in liquid nitrogen or fixed in 4% formalin immediately. For the tail vein injection model, 1 × 10^6^ Luc2-RBE cells stably expressing luciferase and infected with pLKO.1-pre-122 or pLKO.1-NC were injected into mice by tail vein injection. In vivo imaging was performed on a IVIS® Lumina III. AkaLumine, a substrate for firefly luciferase, was injected intraperitoneally (100 μL AkaLumine at 15 mM, MeisenCTCC.Inc) 10 min before luminescence imaging. After injection, mice were anesthetized with 3% isoflurane in an induction chamber and then placed in a Sealed Optical Imaging (OI) Tray, ventilated with 2.5% isoflurane. All animal studies were conducted according to the Association for the Assessment and Accreditation of Laboratory Animal Care and the Institutional Animal Care and Use Committee guidelines.

### Statistical analysis

The results are expressed as the mean ± standard error of the mean. Student’s *t*-test was used for comparison between two groups. One-way analysis of variance was used for comparisons among three or more groups. Tukey’s test, Bonferroni’s test, or Dunnett’s test was used for post hoc multiple comparisons between groups. To analyze tissue microarrays, Fisher’s exact test was used. The level of significance was *p* < 0.05. The number of independent experiments was three (if not depicted otherwise). Calculations were performed using GraphPad Prism (GraphPad, San Diego, CA, USA).

## Results

### miR-122-5p is associated with invasion in ICC and is downregulated in tumors

**Fig. 1:**
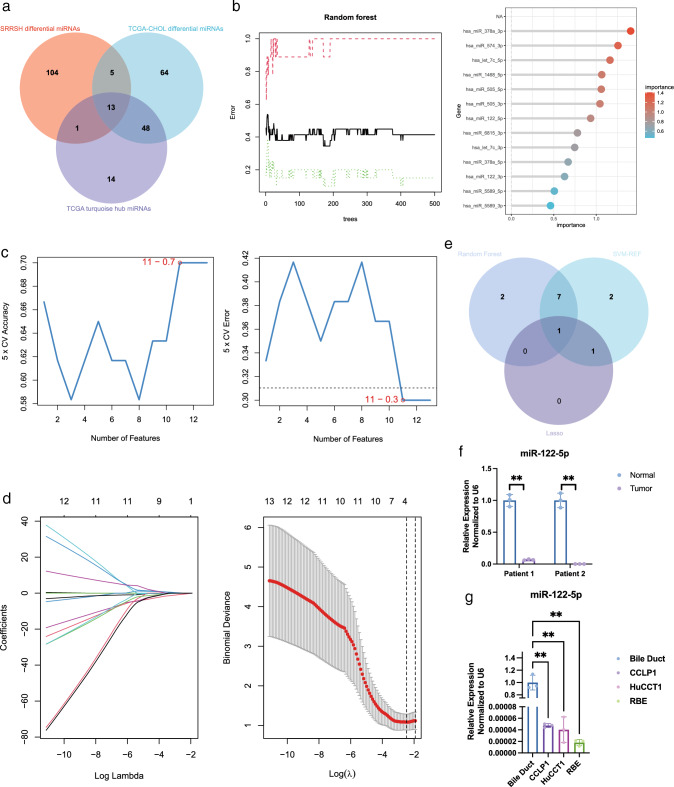
miR-122-5p is associated with invasion in intrahepatic cholangiocarcinoma and is decreased in tumor. **a** Venn diagram of turquoise module hub genes, TCGA differential miRNAs and SRRSH differential miRNAs. **b** Random Forest of 13 filtered miRNAs. **c** SVM-REF of 13 filtered miRNAs. **d** Lasso regression of 13 filtered miRNAs. **e** Venn diagram of three machine learning algorithms including Lasso regression, Random Forest, and SVM-REF. **f** miR-122-5p expression level in 2 pairs of ICC and normal tissue. **g** miR-122-5p expression level in ICC cell lines

To elucidate the potential molecular mechanism underlying the recurrence and early metastasis of ICC, we downloaded TCGA-CHOL miRNA data, used the WGCNA algorithm to separate all miRNAs into nine major modules, and performed correlation analysis of these modules with clinical information related to metastasis. We found that that the turquoise module was significantly negatively associated with neural invasion and tumor recurrence (Fig. S1A–D). In addition, we collected pairs of tumor and normal tissues from 13 patients (SRRSH samples) and performed whole-transcriptome sequencing on these samples, identifying 72 significantly upregulated and 82 significantly downregulated miRNAs (*p* < 0.05, fold change > 1.5) in ICC. We used the intersection of the turquoise module hub miRNAs, differentially expressed miRNAs in TCGA, and differentially expressed miRNAs in SRRSH samples and identified 13 core miRNAs associated with invasion in ICC (Figs. 1a; S2A, B). Then, we used three machine-learning algorithms, namely Lasso regression, random forest, and SVM-REF, to narrow the core miRNAs and finally obtained miR-122-5p (Fig. 1b–e). In addition, we used TCGA database to verify the differences of miR-122-5p expression between cholangiocarcinoma and other tumor types and found that miR-122-5p has the most significant difference in expression in cholangiocarcinoma [28] (Fig. S2C, D).

To further verify the reliability of the sequencing results, we further collected two pairs of ICC and normal tissue and revealed that miR-122-5p was consistently downregulated in tumors (Fig. 1f). Likewise, miR-122-5p was downregulated in ICC cell lines compared with its expression in normal bile duct tissue, in accordance with previous findings [29, 30] (Fig. 1g). Furthermore, tumors with positive lymph node metastasis had lower miR-122-5p expression, suggesting that miR-122-5p is related to ICC metastasis (Fig. S1E). We also tested the efficacy of the 13 hub miRNAs screened for predicting perineural invasion and found that the area under the curve of miR-122-5p was greater than 0.65, ranking third among the examined miRNAs (Fig. S3).

### miR-122-5p inhibits the invasion and migration of ICC in vitro

**Fig. 2:**
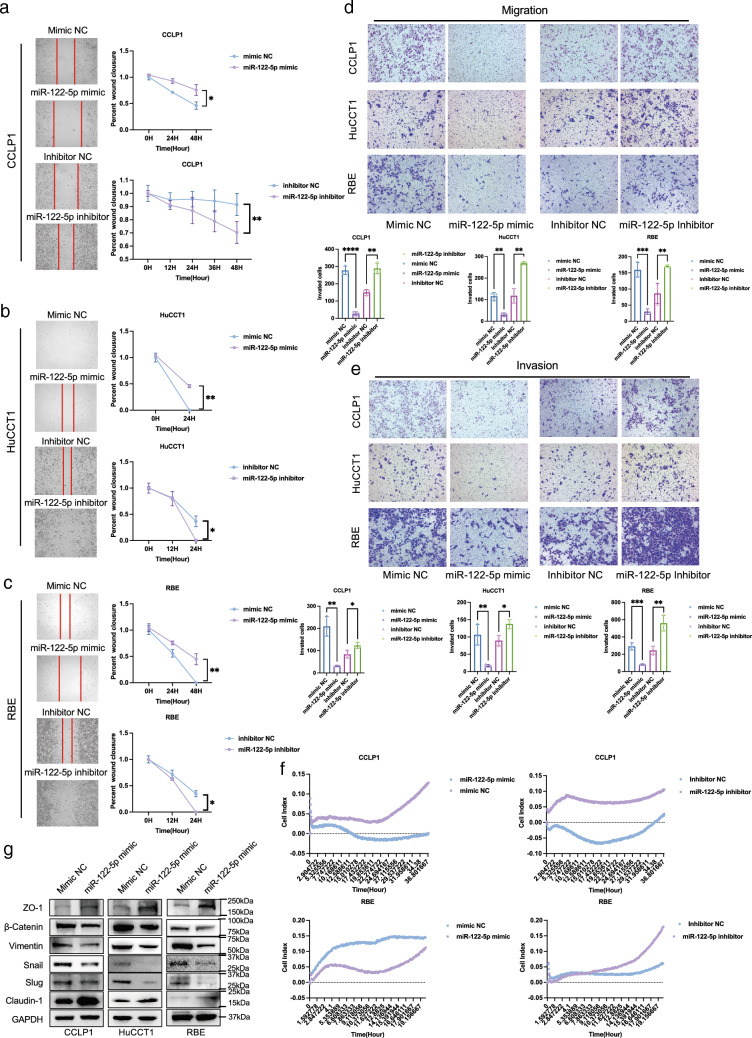
miR-122-5p inhibits invasion and migration of intrahepatic cholangiocarcinoma in vitro*.* **a** Wound healing assay of migration of CCLP1 cells transfected with mimic NC, miR-122-5p mimic, Inhibitor NC, miR-122-5p inhibitor (The magnification is 100×). **b** Wound healing assay of migration of HuCCT1 cells transfected with mimic NC, miR-122-5p mimic, Inhibitor NC, miR-122-5p inhibitor (The magnification is 100×). **c** Wound healing assay of migration of RBE cells transfected with mimic NC, miR-122-5p mimic, Inhibitor NC, miR-122-5p inhibitor (The magnification is 100×). **d** Transwell assay of migration of CCLP1, HuCCT1, RBE cells transfected with mimic NC, miR-122-5p mimic, Inhibitor NC, miR-122-5p inhibitor (The magnification is 100×). **e** Matrigel-based Transwell assay of invasion of CCLP1, HuCCT1, RBE cells transfected with mimic NC, miR-122-5p mimic, Inhibitor NC, miR-122-5p inhibitor (The magnification is 100×). **f** Real-time cellular analysis showed CCLP1 and RBE cells which transfected with miR-122-5p mimics had weaken invasion ability than control cells and which transfected with miR-122-5p inhibitor had better invasion ability than control cells. **g** Western blotting analysis of EMT associated protein level in CCLP1, HuCCT1, RBE cells transfected with miR-122-5p

To further explore the biological function of miR-122-5p in ICC, we first examined the transfection efficiency of miR-122-5p mimic in CCLP1, HuCCT1, and RBE ICC cells (Fig. S4A). The wound-healing assay illustrated that overexpression of miR-122-5p inhibited the migration of ICC cell lines, whereas miR-122-5p inhibition promoted migration (Fig. 2a–c). The Transwell assay further demonstrated that overexpression of miR-122-5p inhibited the migration and invasion of ICC cell lines, whereas miR-122-5p inhibition promoted ICC cell migration and invasion (Fig. 2d, e). Moreover, real-time cellular invasion monitoring demonstrated that miR-122-5p inhibited ICC invasion, whereas miR-122-5p inhibition promoted ICC invasion (Fig. 2f), consistent with the invasive behavior at a fixed time. The CCK-8 assay revealed that miR-122-5p overexpression inhibited the proliferation of CCLP1, HuCCT1, and RBE cells, but miR-122-5p inhibition did not induce the opposite effect (Fig. S4B). miR-122-5p mimic also induced significantly higher apoptotic rates in CCLP1 and RBE cells, higher rates of G1 phase arrest in CCLP1 cells, and higher rates of S phase arrest in RBE cells (Fig. S4D, E). With the inconsistent effects of miR-122-5p on the proliferation and apoptosis of ICC cell lines and the observed effects of miR-122-5p on cell invasion and tumor recurrence, we next focused on its impact on cell migration and invasion. Previous studies demonstrated that EMT was a major factor affecting tumor invasion and metastasis [31]. Thus, we evaluated the expression of EMT-associated proteins and found miR-122-5p inhibited EMT in the aforementioned ICC cell lines (Figs. 2g; S4C).

Together, we demonstrated that miR-122-5p can inhibit the migration and invasion of ICC through a process possibly mediated through the EMT pathway. In addition, the loss of miR-122-5p might be one of the contributors to the increased metastatic and recurrent potential of ICC.

### IGFBP4 is a novel direct target of miR-122-5p

**Fig. 3:**
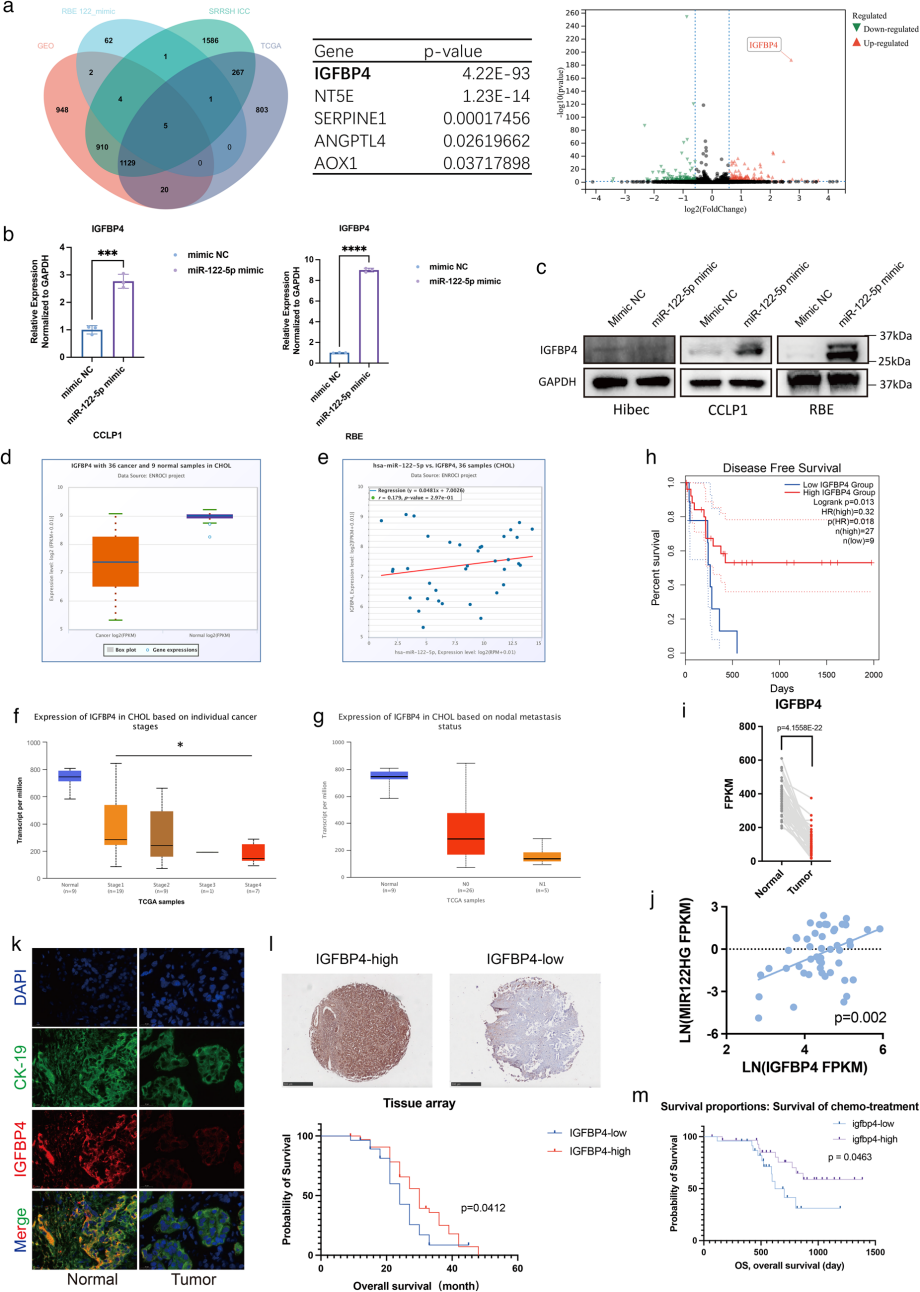
IGFBP4 is a novel direct target of miR-122-5p. **a** Venn diagram of RBE miR-122-5p over expression sequencing data, TCGA differential mRNAs, SRRSH differential mRNAs, GSE138709; list of 5 candidate gene; volcano plot of RBE miR-122-5p over expression sequencing data. **b** IGFBP4 mRNA level in CCLP1, RBE cells transfected with mimic NC, miR-122-5p mimic. **c** Western blotting analysis of IGFBP4 proteins levels in Hibec, CCLP1 and RBE cells transfected with mimic NC, miR-122-5p mimic. **d** Differential expression of IGFBP4 in CCA samples in TCGA databases. The abscissa represents sample type and the ordinate represents gene expression. The orange box indicates tumor sample and purple box indicates normal sample (*p* < 0.05). **e** Positive correlation between miR-122-5p and IGFBP4 in the TCGA CHOL dataset (*R* = 0.179, *p* = 2.79e-01). **f** Differential expression of IGFBP4 on individual cancer stages in TCGA databases. The abscissa represents individual cancer stages and the ordinate represents gene expression. **g** Differential expression of IGFBP4 on nodal metastasis status in TCGA databases. **h** Kaplan–Meier survival curves of patients with low or high IGFBP4 protein expression based on TCGA database. Significance was determined using Kaplan–Meier analyses. Survival analysis was performed using log-rank test. **i** Differential expression of IGFBP4 in CCA samples in SRRSH databases. The abscissa represents sample type and the ordinate represents gene expression. The red dots indicates tumor sample and grey dots indicates normal sample (*p* = 4.1558E-22). **j** Positive correlation between miR-122-5p and IGFBP4 in the SRRSH dataset (*p* = 0.002). **k** Representative mIHC images of IGFBP4 staining in tumors and normal. **l** Representative images of IGFBP4 staining in tissue array. Kaplan–Meier survival curves of patients with low or high IGFBP4 protein expression based on tissue array. Significance was determined using Kaplan–Meier analyses. Survival analysis was performed using log-rank test. **m** Kaplan–Meier survival curves of patients with low or high IGFBP4 protein expression based on Fanjia’s database. Significance was determined using Kaplan–Meier analyses. Survival analysis was performed using log-rank test

To explore the specific mechanism through which miR-122-5p inhibited the invasion and migration of ICC cells, we performed transcriptome analysis of RBE cells transfected with miR-122-5p mimic and negative control. Through sequencing analysis, we identified 168 upregulated miRNAs (fold change > 1) and 174 downregulated miRNAs (fold change > 1) with statistical significance (*p* < 0.05) in the miR-122-5p mimic group compared with the findings in the negative control group. We took the intersection of the genes of previous sequencing data, differentially expressed mRNAs in TCGA, differentially expressed mRNAs in SRRSH samples, and GSE138709 data and identified five core mRNAs associated with miR-122-5p. Among them, IGFBP4 was the most significantly regulated gene, and it was upregulated upon miR-122-5p overexpression (Fig. 3a). RT-qPCR further confirmed the sequencing results in CCLP1 and RBE cells, revealing the upregulation of IGFBP4 mRNA following transfection with miR-122-5p mimic (Fig. 3b). miR-122-5p overexpression in ICC cell lines also increased the protein levels of IGFBP4, while similar phenomenon did not occur in hibec cells (Fig. 3c). TCGA database analysis found that IGFBP4 was expressed at a lower level in ICC tumors, and the mRNA levels of IGFBP4 were positively correlated with those of miR-122-5p (Fig. 3d, e). In-depth analysis of TCGA database further revealed that IGFBP4 expression gradually decreased with the progression of ICC, and in patients with lymph node metastases, IGFBP4 appears to be expressed at lower levels (Fig. 3f, g). TCGA data also demonstrated that patients with lower IGFBP4 expression had lower disease-free survival (Fig. 3h). To validate these findings, we collected patients with ICC from 2008 to 2019 who underwent surgery in Sir Run Run Shaw Hospital (*N* = 53) for transcriptome analyses and found decreased levels of IGFBP4, which were positively correlated with miR-122-5p levels (Fig. 3i, j). In addition, multiplex immunohistochemistry (mIHC) and immunofluorescence analysis of paired ICC and normal tissues revealed lower protein IGFBP4 expression in ICC (Fig. 3k, Figure S5A). In addition, we also performed tissue microarray and found that the high IGFBP4 expression group had significantly better prognosis (*p* < 0.05, Fig. 3l).

Together, we illustrated that IGFBP4 is expressed at a significantly lower level in tumors and is positively correlated with miR-122-5p.

### IGFBP4 inhibits the invasion and migration of ICC in vitro

**Fig. 4:**
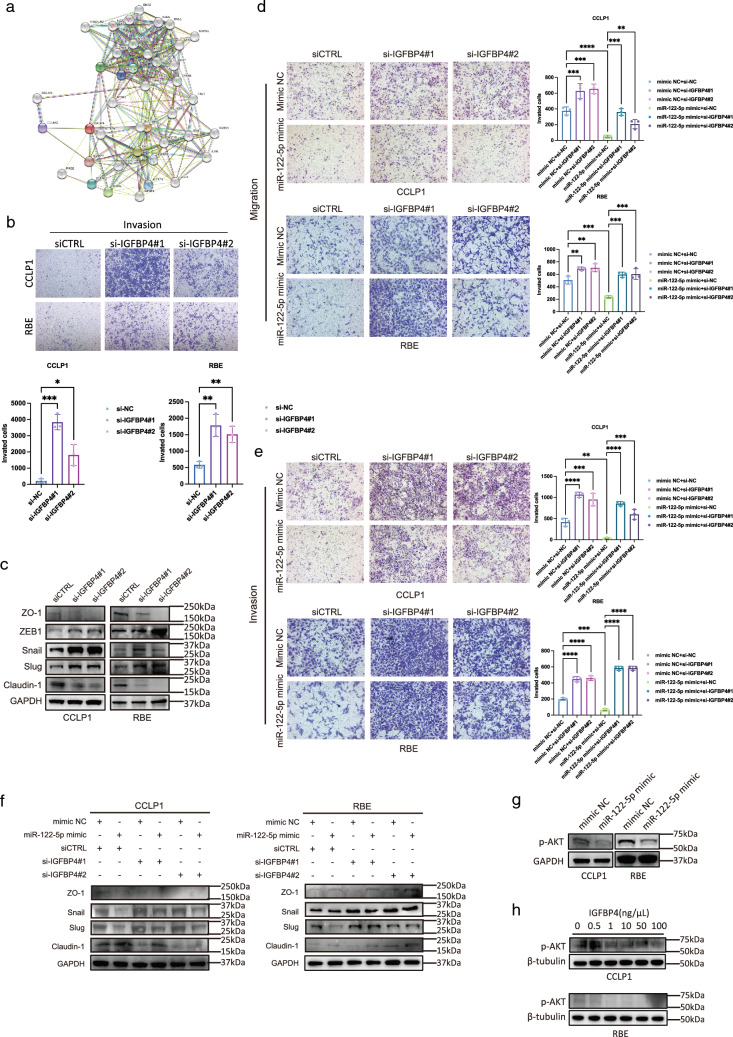
IGFBP4 inhibits invasion and migration of intrahepatic cholangiocarcinoma in vitro. **a** Protein–protein interaction (PPI) analysis of IGFBP4. **b** Matrigel-based Transwell assay of invasion of CCLP1, RBE cells transfected with si-NC, si-IGFBP4#1, si-IGFBP4#2 (The magnification is 100×). **c** Western blotting analysis of EMT associated protein level in CCLP1, RBE cells transfected with si-NC, si-IGFBP4#1, si-IGFBP4#2. **d** Transwell assay of migration of CCLP1, RBE cells transfected with si-NC, si-IGFBP4#1, si-IGFBP4#2, mimic NC, miR-122-5p (The magnification is 100×). **e** Matrigel-based Transwell assay of invasion of CCLP1, RBE cells transfected with si-NC, si-IGFBP4#1, si-IGFBP4#2, mimic NC, miR-122-5p (The magnification is 100×). **f** Western blotting analysis of EMT associated protein level in CCLP1, RBE cells transfected with si-NC, si-IGFBP4#1, si-IGFBP4#2, mimic NC, miR-122-5p. **g** Western blotting analysis of p-AKT proteins levels in CCLP1 and RBE cells transfected with mimic NC, miR-122-5p mimic. **h** Western blotting analysis of p-AKT proteins levels in CCLP1 and RBE cells treated with different concentration of IGFBP4 protein

After identifying IGFBP4 as a likely downstream target of miR-122-5p, we next verified its function in ICC. Protein–protein interaction analysis suggested that IGFBP4 is associated with IGF1, IGF2, and extracellular matrix associated proteins such as COL4A2, which was a significant factor affecting tumor metastasis and implying that IGFBP4 affects cell invasion through the IGF signaling pathway (Fig. 4a). Indeed, Transwell assays demonstrated that silencing IGFBP4 promoted the migration and invasion of ICC cells (Fig. 4b). Meanwhile, silencing IGFBP4 also promoted EMT in ICC cells (Figs. 4c; S5B–D). To verify whether the inhibitory effects of miR-122-5p on the invasion and metastasis of cholangiocarcinoma occurs through the regulation of IGFBP4, we performed rescue assays. The results demonstrated that silencing IGFBP4 attenuated the inhibitory effect of miR-122-5p on invasion and migration in ICC cells (Fig. 4d, e). Western blotting also indicated that silencing IGFBP4 could undermine the effects of miR-122-5p on MET markers, especially SLUG (Figs. 4f; S5E). Notably, our analysis of data from Fudan University revealed that high IGFBP4 expression was a protective factor for tumor chemotherapy, implying that IGFBP4 can be used to guide postoperative chemotherapy [32] (Fig. 3m).

Together, we found that the inhibition of ICC invasion and metastasis by miR-122-5p might be mediated through IGFBP4.

### IGFBP4 inhibits EMT of ICC by suppressing the IGF1R pathway and AKT phosphorylation

It is generally accepted that IGFBP4 inhibits tumor development and progression by sequestering IGFs [27]. Previous studies reported that IGFBP4 can inhibit the IGF1R pathway [33, 34] and AKT phosphorylation to suppress tumor growth [26], and p-AKT protein was identified as one of the proteins regulating EMT. Therefore, we speculated that in ICC, the inhibitory effect of IGFBP4 on EMT was also achieved by inhibiting the IGF1R pathway. Using western blotting, we found that overexpression of miR-122-5p could suppress the intracellular p-AKT level (Fig. 4g). When we added soluble IGFBP4 protein to the culture medium, the p-AKT level gradually decreased as the mount of the exogenous protein was increased (Fig. 4h).

These experiments demonstrated that miR-122-5p could inhibit the phosphorylation of AKT by promoting IGFBP4 expression, thereby inhibiting EMT of ICC.

### miR-122-5p binds to the promoter region of IGFBP4 and activates its transcription

**Fig. 5:**
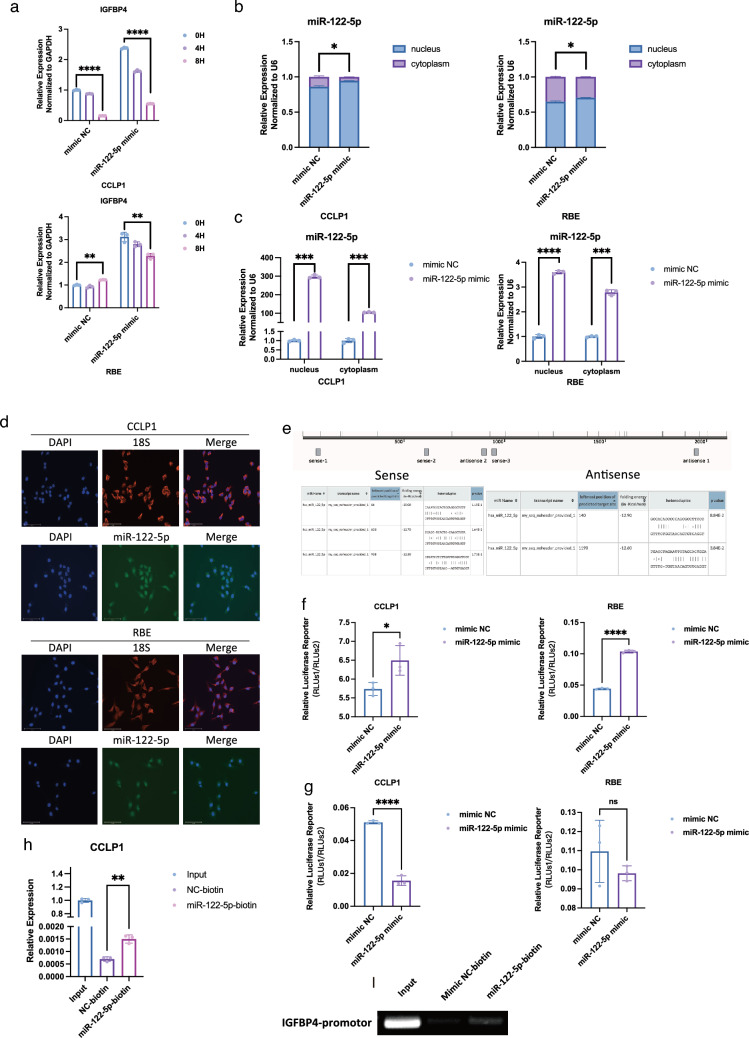
miR-122-5p binds to the promoter region of IGFBP4 and activates its transcription. **a** Time-course of relative IGFBP4 expression in CCLP1 and RBE cells treated with actinomycin d (10 μg/mL). **b** Relative miR-122-5p of nucleus and cytoplasm in CCLP1 and RBE cells transfected with mimic NC, miR-122-5p mimic. **c** Absolute miR-122-5p of nucleus and cytoplasm in CCLP1 and RBE cells transfected with mimic NC, miR-122-5p mimic. **d** Localization of miR-122-5p (green) in CCLP1 and RBE cells using fluorescence in situ hybridization. Cell nuclei were counterstained with DAPI (blue). Scale bar, 100 μm. **e** Predicted binding site of miR-122-5p on promotor region of IGFBP4. **f** Dual Renilla and firefly luciferase reporter assays using the linear form of wild-type IGFBP4 promotor in CCLP1 and RBE cells transfected with miR-122-5p mimics. **g** Dual Renilla and firefly luciferase reporter assays using the linear form of mutant IGFBP4 promotor in CCLP1 and RBE cells transfected with miR-122-5p mimics. **h** qPCR revealed miR-122-5p could bind to promotor region of IGFBP4. **i** ChiRP assay revealed miR-122-5p could bind to promotor region of IGFBP4

To explore the detailed mechanisms by which miR-122-5p regulates IGFBP4 mRNA levels, we first detected the effect of miR-122-5p on IGFBP4 mRNA stability following actinomycin D treatment. However, the results demonstrated that miR-122-5p did not increase the mRNA stability of IGFBP4 (Fig. 5a). Therefore, we speculated that miR-122-5p might increase IGFBP4 mRNA intracellular content by increasing its production. To test this hypothesis, we first analyzed the intracellular localization of miR-122-5p. The nucleocytoplasmic separation assay and FISH illustrated that intrinsic miR-122-5p was distributed in both the cytoplasm and nucleus, but mainly in the nucleus (Fig. 5b, d). Similarly, both nuclear and cytoplasmic miR-122-5p levels were increased in cells following miR-122-5p overexpression, although the change was greater in the nucleus (Fig. 5b, c). Therefore, these results suggested that miR-122-5p might increase IGFBP4 mRNA levels by activating its transcription. We searched the potential binding sites for miR-122-5p in the IGFBP4 promoter region using online prediction tools [35] and found five potential miR-122-5p binding sites in the promoter region of IGFBP4 (Fig. 5e). Dual-luciferase reporter assays demonstrated that miR-122-5p could enhance the transcription of IGFBP4 through its wild-type promoter (Fig. 5f), whereas this did not occur with the mutated promoter (Figs. 5g, S5F). These results suggested that miR-122-5p could interact with the promoter of IGFBP4 to enhance its transcription. In support of this view, miR-122-5p could directly bind to the promoter region of IGFBP4 in a pulldown assay with biotinylated miR-122-5p through transfection followed by chromatin isolation by RNA purification (ChiRP) (Fig. 5h, i).

Together, we demonstrated that miR-122-5p can directly bind to the promoter region of IGFBP4 and enhance its transcription.

### miR-122-5p inhibits the invasion and migration of ICC in vivo

**Fig. 6:**
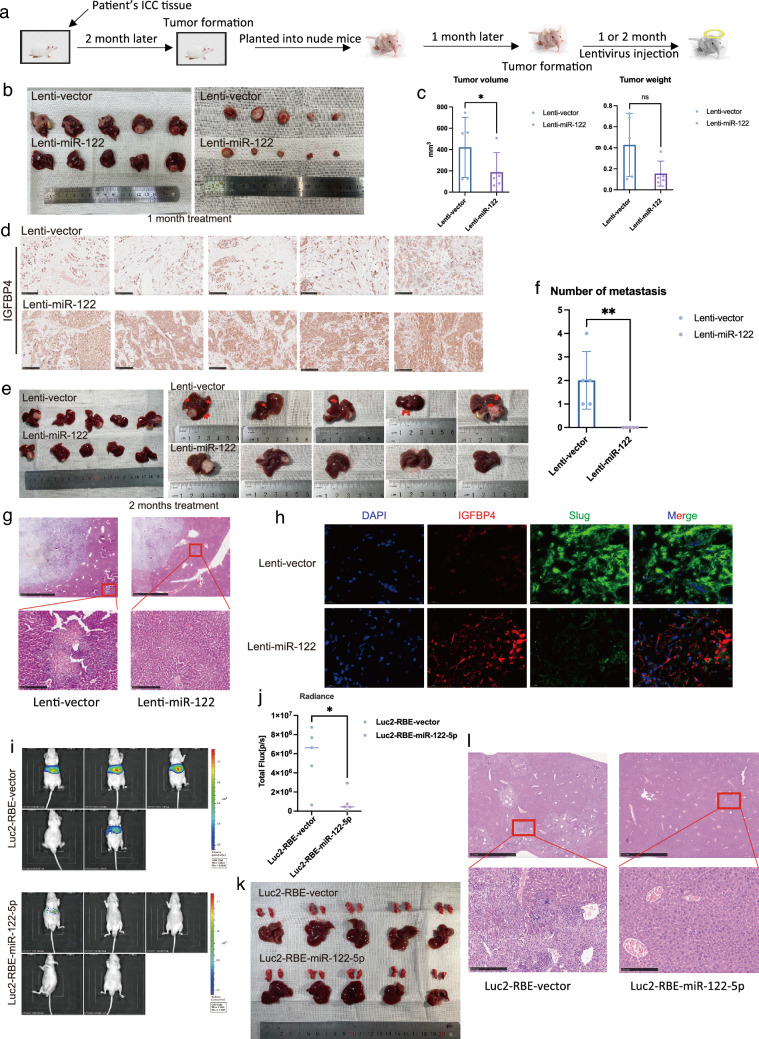
miR-122-5p inhibits invasion and migration of intrahepatic cholangiocarcinoma in vivo*.* **a** Flow Chart of PDX intrahepatic metastasis model construction. The first PDX generation was constructed in 4-week-old male Nod/scid mice. Two months later, the xenograft was disaggregated and implanted subcutaneously into 4–6 week-old BALB/c nude mice as the first PDX. One month after implantation, the first PDX mice were injected with OE-pre-122 lentivirus or its negative control through tail vein (twice a week for 1 or 2 months). Mice were euthanized on the forth week and eighth week and whole liver were isolated and the intrahepatic metastasis were measured. The tumor size was measured and recorded every 2 days using the Vernier caliper as follows: tumor volume (mm^3^) = (*L* × *W*2)/2, where *L* is the long axis and *W* the short axis. **b** Gross view of tumors from two groups; Liver view of tumors from two groups after one month treatment. **c** The tumor weight and volume of tumors from two groups after one month treatment. **d** Immunohistochemistry for IGFBP4 comparing OE- pre-122 and control PDX. Scale bar, 100 μm. **e** Gross view of tumors from two groups; Liver view of tumors from two groups after two months treatment. **f** Number of metastasis from two groups after two month treatment. **g** HE staining comparing OE- pre-122 and control PDX. Scale bar, 100 μm. **h** Representative mIHC images of Dapi\IGFBP4\slug staining in tumors of OE- pre-122 and control PDX. **i** Bioluminescence imaging of in vivo metastatic activity in each group. **j** Statistical analysis of mice with liver metastasis in each group. **k** Gross view of livers and lungs from two groups after three months of tail vein injection. **l** H&E staining showing the metastatic tumours in livers

To examine the antitumor effects of miR-122-5p against ICC in vivo and closely approximate the clinical environment, we chose an orthotopic patient-derived tumor xenograft (PDX) model for in vivo experiments. We first implanted fresh ICC tumor tissue excised from patients into both flanks of NOD/SCID mice. After approximately 2 months of tumor growth, we removed the tumors and re-implanted 1 × 1 mm^2^ portions into the livers of nude mice. After 1 month of tumor growth, we started tail vein injections of miR-122–expressing lentivirus and empty lentivirus control. After another 1 or 2 months of treatment, the mice were sacrificed (Fig. 6a). After 1 month of treatment, tumors in control mice exhibited marked invasive growth patterns, and they invaded other organs such as the stomach and small intestine. Conversely, no metastatic lesions were detected in treated mice (Fig. 6b). After tumor excision, we found that tumor size were significantly lower in the treatment group than in the control group (Fig. 6c). IHC further demonstrated that miR-122-5p lentivirus treatment increased the expression of miR-122-5p and IGFBP4 in the tumor, suggesting that miR-122-5p can also promote the expression of IGFBP4 in vivo (Figs. 6d; S5G). After 2 months of treatment, we found prominent intrahepatic metastasis within the liver of the control group, whereas no metastasis was found in the treatment group (Fig. 6e–g), and protein level of slug was obviously decreased (Fig. 6h). In tail vein injection model, the fluorescence intensity of livers in Luc2-RBE-miR-122-5p were much less than that in Luc2-RBE-vector (Fig. 6i–k). The livers of nude mice were harvested after 3 months injection. Overexpressed miR-122-5p led to less liver metastases (Fig. 6l).

These results demonstrated that miR-122-5p could inhibit the metastasis of ICC in vivo.

**Fig. 7:**
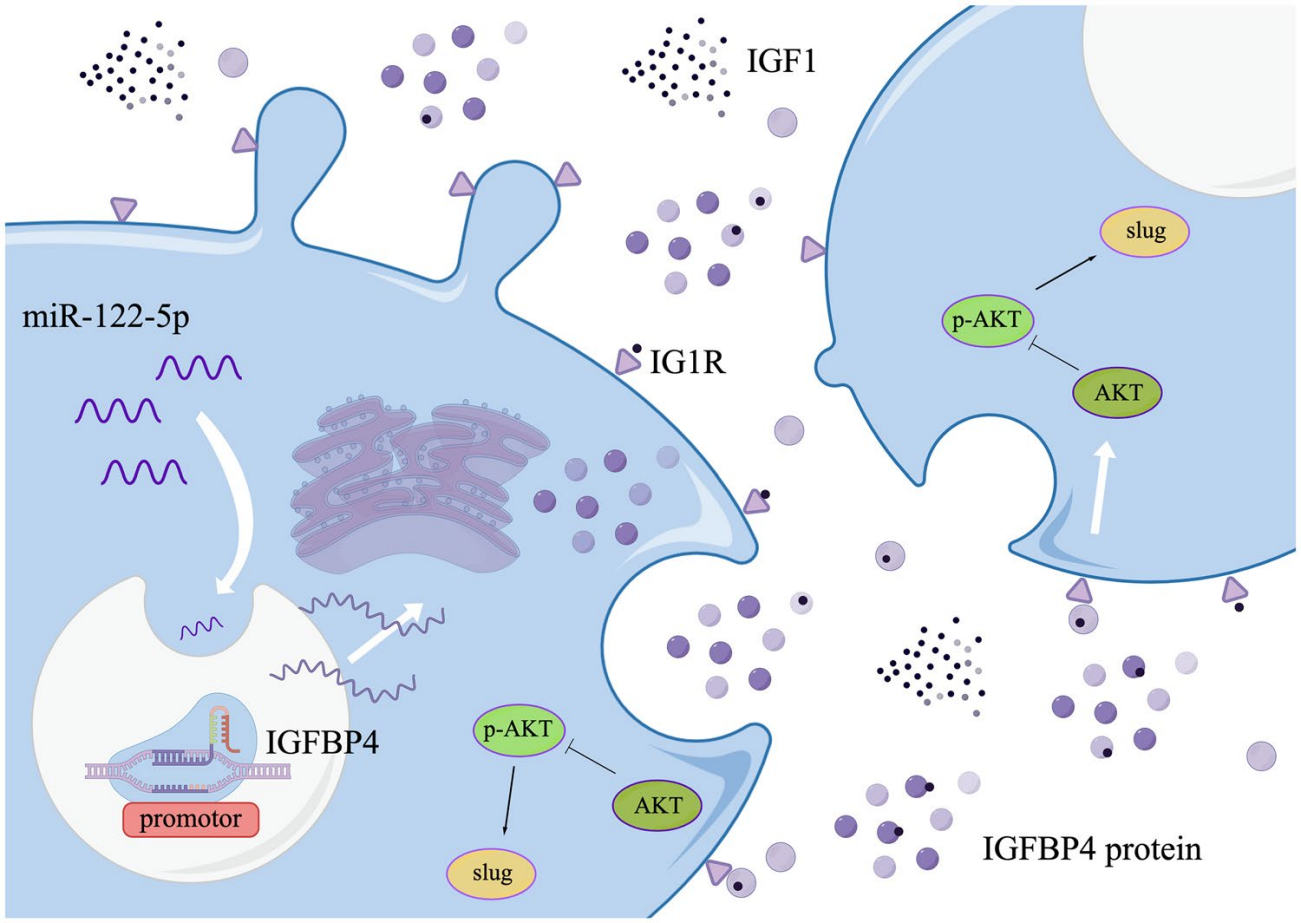
Hypothetical model for miR-122-5p in ICC. **a** Hypothetical model for miR-122-5p in ICC. miR-122-5p acts as transcription activator for IGFBP4 to inhibit ICC invasion by regulating the EMT signaling pathway

Overall, our results revealed a novel mechanism of miR-122-5p activating transcription of IGFBP4 and highlighted the clinical value of miR-122-5p and IGFBP4 in inhibiting ICC invasion and metastasis (Fig. 7a).

## Discussion

ICC is the second most common primary malignancy in the liver, currently accounting for approximately 15% of all primary liver cancers [1], and its incidence is increasing globally [2]. In addition, most patients with ICC are diagnosed with advanced disease, and they have no opportunity for surgery. Although various clinical trials successively demonstrated the efficacy of several targeted drugs and immune checkpoint inhibitors in the treatment of ICC over the past decade, most systemic therapies have not greatly improved patient outcomes. The overall prognosis of ICC remains poor. Therefore, there is an urgent need in clinical practice to explore new therapeutic targets and advance the precision treatment of ICC.

As a liver-specific miRNA, the function of miR-122 in liver pathophysiology has been intensively studied. Wang et al. analyzed circulating miRNAs associated with drug-induced liver injury and found an average 470-fold increase in miR-122 expression in plasma samples with excessive acetaminophen content [36]. miR-122 may also be involved in the pathogenesis of chronic hepatitis C by promoting viral replication and liver tropism at the translational level by increasing the association of ribosomes with viral RNA during early initiation [37, 38]. Tsai et al. found that miR-122 was significantly downregulated in intrahepatic metastatic HCC, and mice lacking the gene encoding miR-122a develop non-alcoholic steatohepatitis, fibrosis, and HCC [39]. Although the research on miR-122 in HCC is relatively in-depth, its role in ICC has not been extensively studied. The existing studies on ICC and miR-122 only demonstrated that miR-122 can inhibit ICC proliferation and metastasis by targeting ALDOA and chloride intracellular channel 1. However, it is unclear whether miR-122 is effective in animals [40, 41]. As for the reason why miR-122-5p is downregulated in ICC, there have been some reports. Sun et al. found that ERK activation suppressed nuclear export activity of XPO5 which downregulated miR-122-5p expression [42]. Loss of liver-enriched transcription factor such as HNF1A, HNF3A, HNF3B, HNF4A, HNF4G, and HNF6 could also induce the loss of miR-122-5p [43, 44]. In addition, long non-coding RNA like UCA1 and PCAT1 have been reported acting as miRNA sponge, which downregulated miR-122-5p expression [41, 45].

In our experiment, we screened miR-122-5p using the sequencing data of patients with ICC, and proved that miR-122-5p has inhibitory effects on ICC invasion and metastasis using comprehensive experimental approaches including wound-healing experiments, Transwell, and real-time label-free experiments.

Conversely, the conventional mechanism of by which miR-122-5p suppresses 3′UTR failed to account for its effects on targets identified through unbiased analysis of the transcriptomes of cells expressing miR-122-5p. We discovered a potential novel regulatory mechanism whereby miR-122-5p transcriptionally activates IGFBP4 expression. Our study uncovered a rare mechanism of miR-122-5p and provided a new possibility to study the function of miR-122-5p in the future.

Previous models of invasion and metastasis used gene-modified cells for tail vein or splenic vein injection to observe the lung or liver metastasis of mice to determine whether the treatment was effective, but such models cannot completely simulate the transfer mode of ICC in the human body. Because the injection model using the tail vein and spleen vein starts directly from the spread of tumor cells into blood, lacking the process of tumor cell extravasation through the basement membrane and into blood vessels, which is a significant step in tumor metastasis. Moreover, cellular experiments cannot simulate the heterogeneity of ICC. In our study, an orthotopic PDX model was used to simulate the actual situation of ICC metastasis in the human body as much as possible, and PDX could better simulate the heterogeneity of ICC. The safety and efficacy of miR-122-5p treatment were further confirmed via tail vein injections of pre-miR-122 lentivirus solution.

Our study had some limitations. We did not characterize why miR-122-5p was specifically downregulated in ICC tumors as well as the implication of the unique regulation of IGFBP4 by miR-122-5p. Although previous literature reported that IGFBP4 can inhibit the IGF1R pathway by binding to IGF1, we did not provide a clear demonstration. Nevertheless, our findings provide a solid foundation to expand the mechanistic understanding of miRNA in ICC in addition to its potential clinical utility in treating ICC.

## Conclusion

Our study revealed a novel mechanism of miR-122-5p by which it can directly bind to the promotor region of IGFBP4 activating its transcription and attenuate the metastasis of intrahepatic cholangiocarcinoma. We also highlighted the clinical value of targeting miR-122-5p and IGFBP4 in inhibiting ICC invasion and metastasis.

### Supplementary Information

Below is the link to the electronic supplementary material.

Figure S1 Supplementary data for miR-122-5p is associated with invasion in intrahepatic cholangiocarcinoma and is decreased in tumor. (A) miRNA dendrogram showing the co-expression modules. (B) Relationships of consensus MEMs and clinical traits. (C) The plot shows the scale-free topology fit index (y-axis) for different soft-thresholding powers (β) (x-axis). Analysis of the mean connectivity (degree, y-axis) for various soft-thresholding powers (x-axis). (D) Module Eigenvector Clustering of 9 modules. Figure S2 Supplementary data for miR-122-5p is associated with invasion in intrahepatic cholangiocarcinoma and is decreased in tumor.(A) Volcano plot of miRNA in SRRSH ICC database. (B) Volcano plot of miRNA in TCGA CHOL database. (C) miR-122-5p is also down regulated in other types of cancer in TCGA database. (D) Differential expression of miR-122-5p in CCA samples in TCGA databases. The abscissa represents sample type and the ordinate represents gene expression. The orange box indicates tumor sample and purple box indicates normal sample (P < 0.05). (E) Differential expression of miR-122-5p on nodal metastasis status in TCGA databases. Figure S3 The efficacy of 13 hub miRNAs in diagnosing perineural invasion. (A) The efficacy of miR-122-5p in diagnosing perineural invasion. (B) The efficacy of Let-7c-3p in diagnosing perineural invasion. (C) The efficacy of Let-7c-5p in diagnosing perineural invasion. (D) The efficacy of miR-122-3p in diagnosing perineural invasion. (E) The efficacy of miR-378a-3p in diagnosing perineural invasion. (F) The efficacy of miR-378a-5p in diagnosing perineural invasion. (G) The efficacy of miR-505-3p in diagnosing perineural invasion. (H) The efficacy of miR-505-5p in diagnosing perineural invasion. (I) The efficacy of miR-574-3p in diagnosing perineural invasion. (J) The efficacy of miR-1468-5p in diagnosing perineural invasion. (K) The efficacy of miR-5589-3p in diagnosing perineural invasion. (L) The efficacy of miR-5589-5p in diagnosing perineural invasion. (M) The efficacy of miR-6815-3p in diagnosing perineural invasion. Figure S4 miR-122-5p inhibits proliferation, cell cycle, activate apoptosis of intrahepatic cholangiocarcinoma in vitro. (A) The expression of miR-122-5p in CCLP1, HuCCT1, RBE cells transfected with miR-122-5p mimic or mimic NC. (B)CCK-8 analysis and representative results of CCLP1, HuCCT1, RBE cells transfected with miR-122-5p mimic or mimic NC and miR-122-5p inhibitor or inhibitor NC. (C) Western blotting analysis of EMT associated protein level in CCLP1, HuCCT1, RBE cells transfected with miR-122-5p. (D)Apoptosis analysis and representative results of CCLP1, HuCCT1, RBE cells using flow cytometry transfected with miR-122-5p mimic or mimic NC. (E) Cell cycle analysis and representative results of CCLP1, RBE cells using flow cytometry transfected with miR-122-5p mimic or mimic NC. Figure S5 Supplementary data for IGFBP4. (A) Representative immunofluorescence images of IGFBP4 staining in tumors and normal. (B) The expression of IGFBP4 in CCLP1, RBE cells transfected with si-NC, si-IGFBP4#1, si-IGFBP4#2. (C) Western blotting analysis of IGFBP4 proteins levels in CCLP1 and RBE cells transfected with si-NC, si-IGFBP4#1, si-IGFBP4#2. (D) Western blotting analysis of EMT associated protein level in CCLP1, RBE cells transfected with si-NC, si-IGFBP4#1, si-IGFBP4#2. (E) Western blotting analysis of EMT associated protein level in CCLP1, RBE cells transfected with si-NC, si-IGFBP4#1, si-IGFBP4#2, mimic NC, miR-122-5p.(F) Sequence of wild type IGFBP4 promotor region and mutant IGFBP4 promotor region. (G) The expression of miR-122-5p in PDX tumor via tail vein injection of miR-122–expressing lentivirus and empty lentivirus control. (H) Representative HE staining images comparing OE- pre-122 and control PDX. Scale bar, 100 μm. Supplementary file1 (PDF 4927 KB)

Supplementary file2 (XLSX 9 KB)

Supplementary file3 (XLSX 10 KB)

Supplementary file4 (XLSX 9 KB)

## Data Availability

Additional data collected during this study are available from the corresponding authors upon reasonable request.
