# Supplementary figures and images for: Activation of IGFBP4 via unconventional mechanism of miRNA attenuates metastasis of intrahepatic cholangiocarcinoma
Source: Hepatol Int. 2023 Jun 22;18(1):91–107. doi: 10.1007/s12072-023-10552-7 (PMC10858123; doi:10.1007/s12072-023-10552-7)

Figure S1

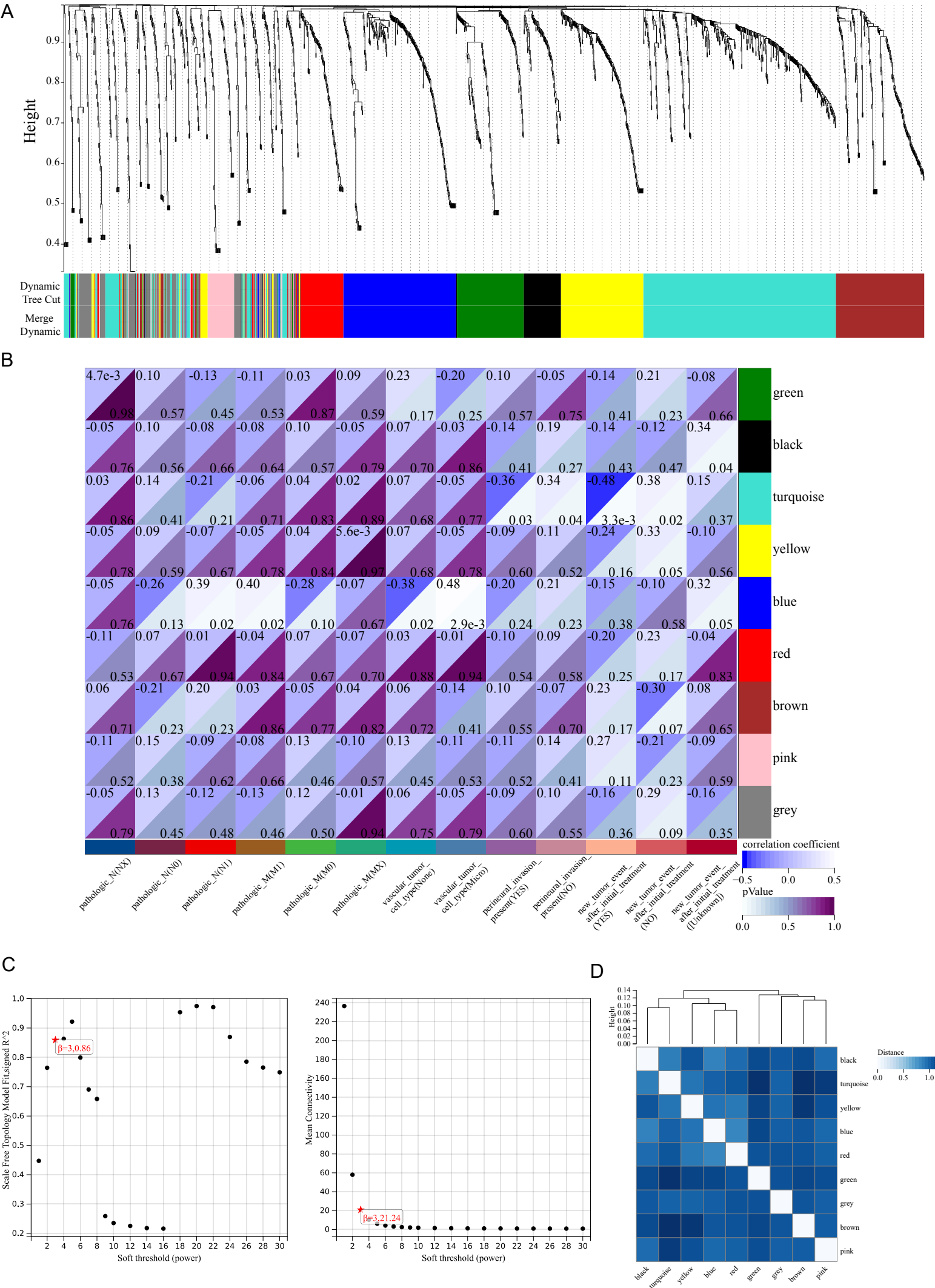

Figure S2

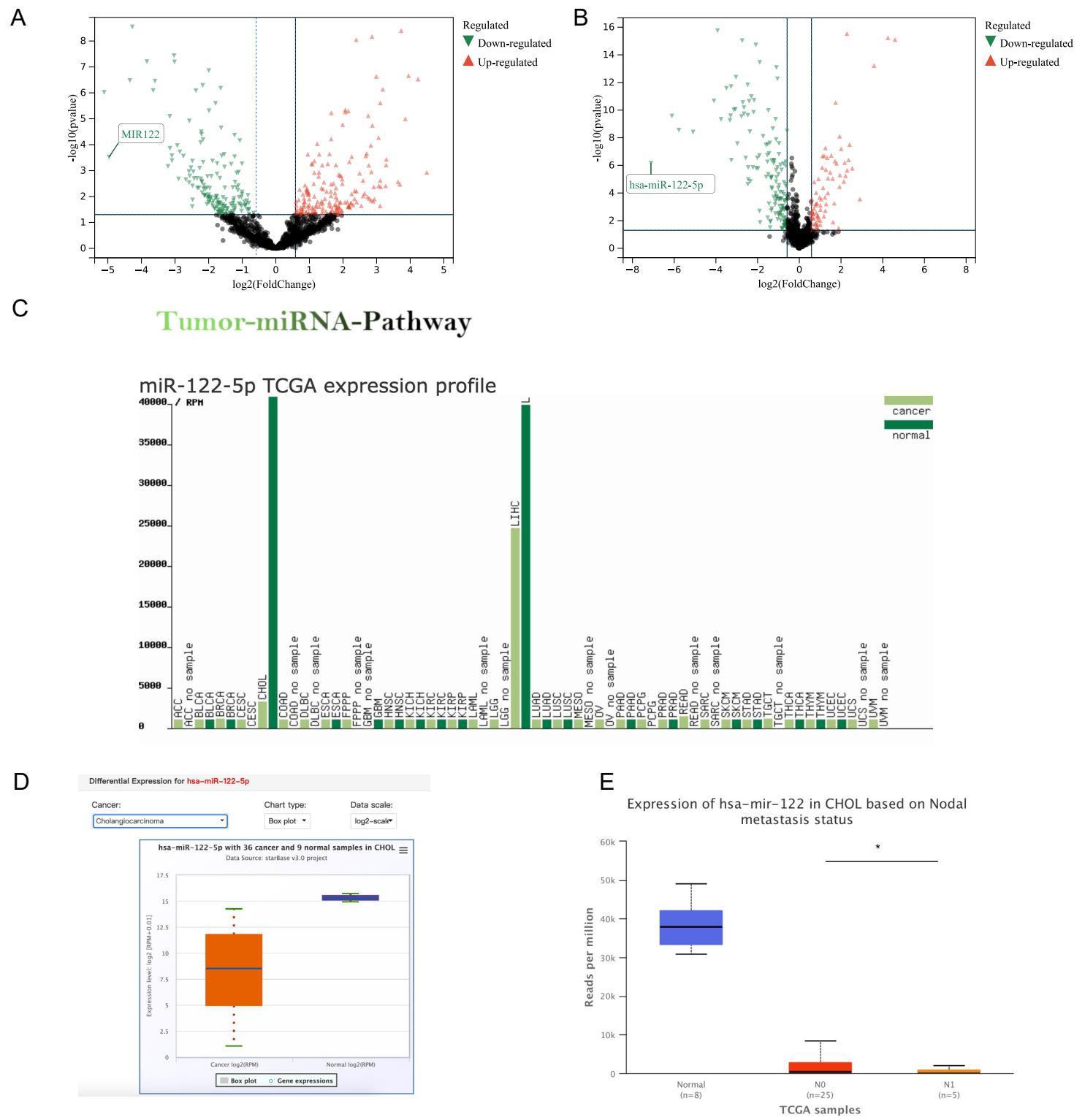

Figure S3

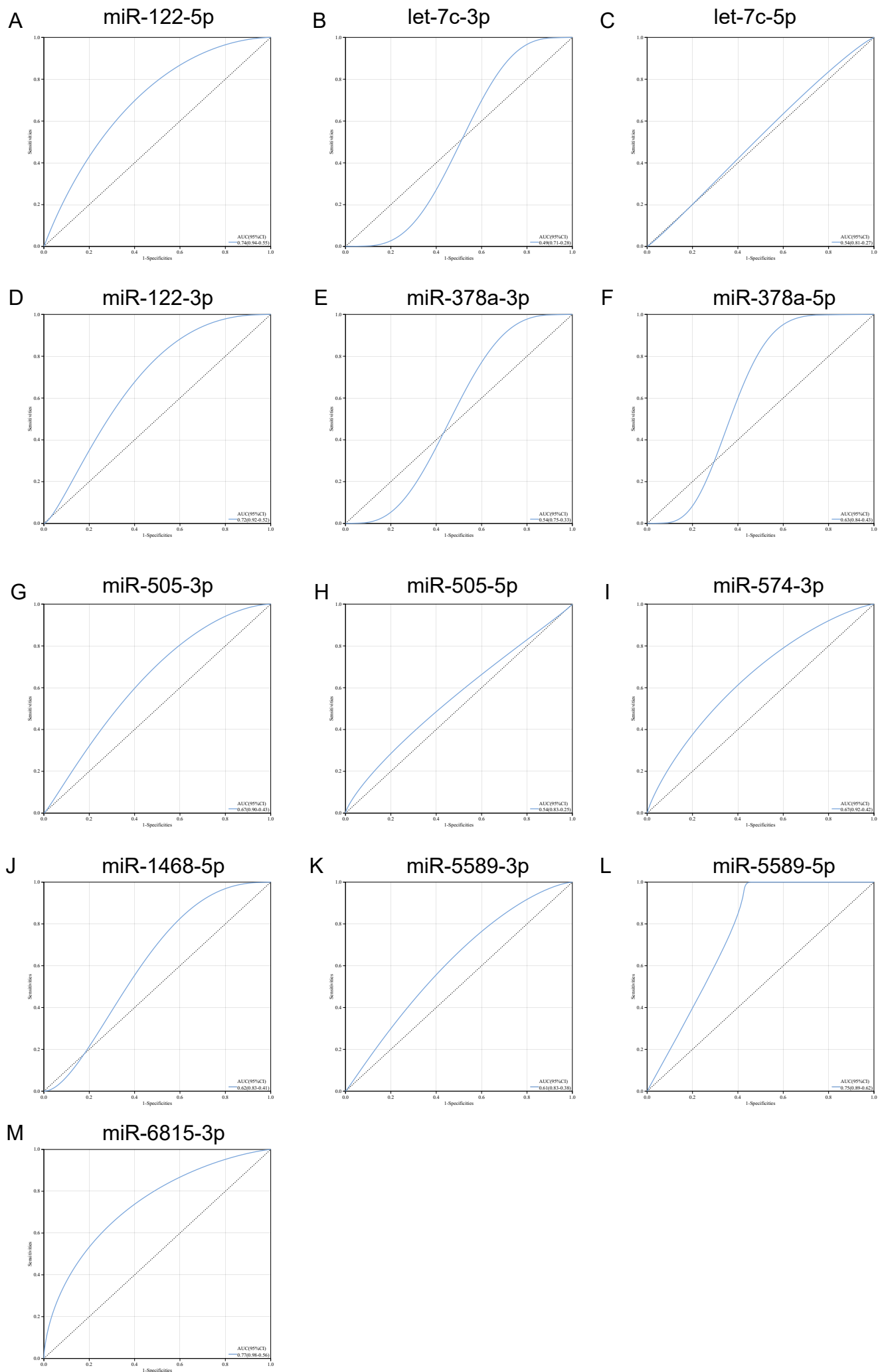

Figure S4

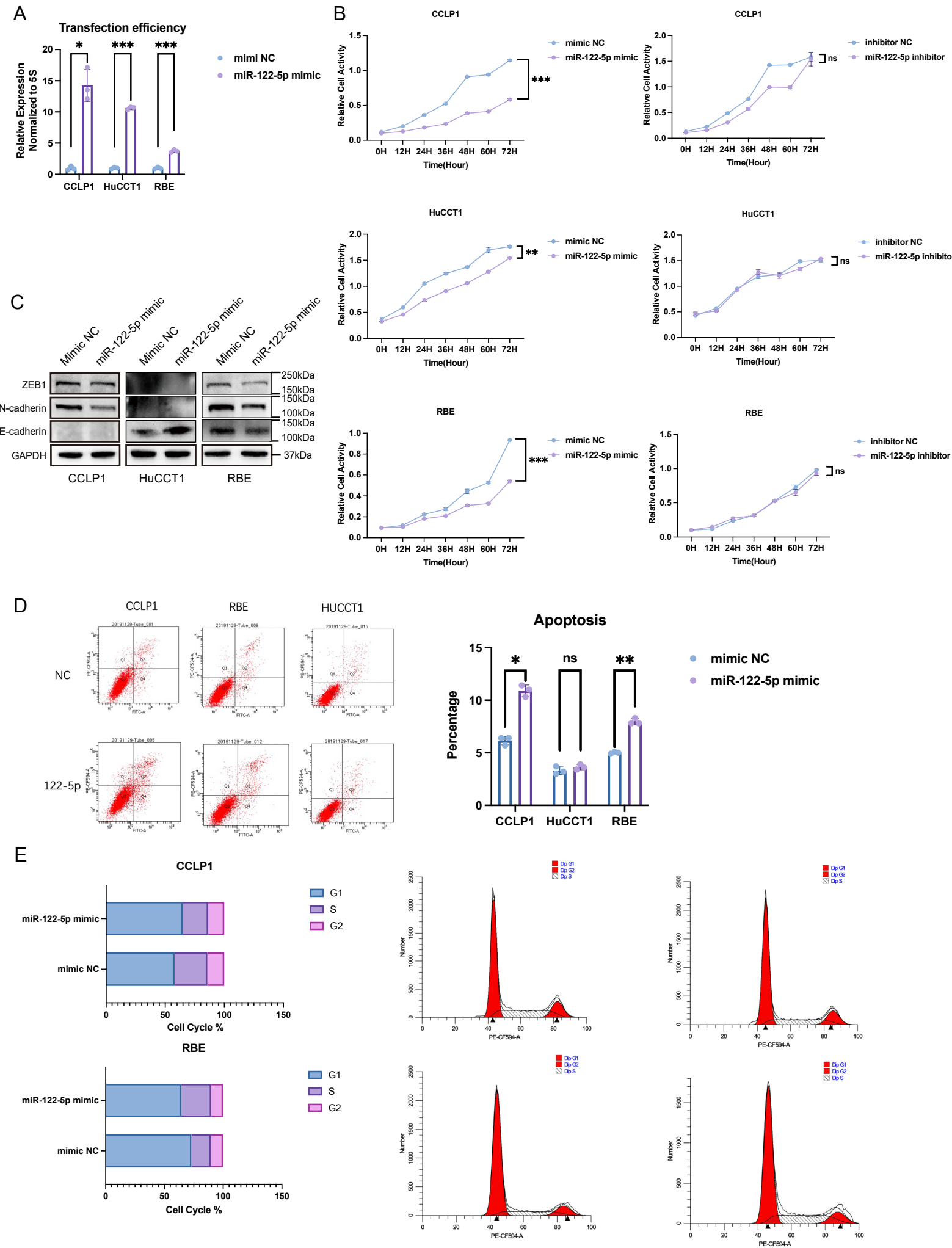

Figure S5

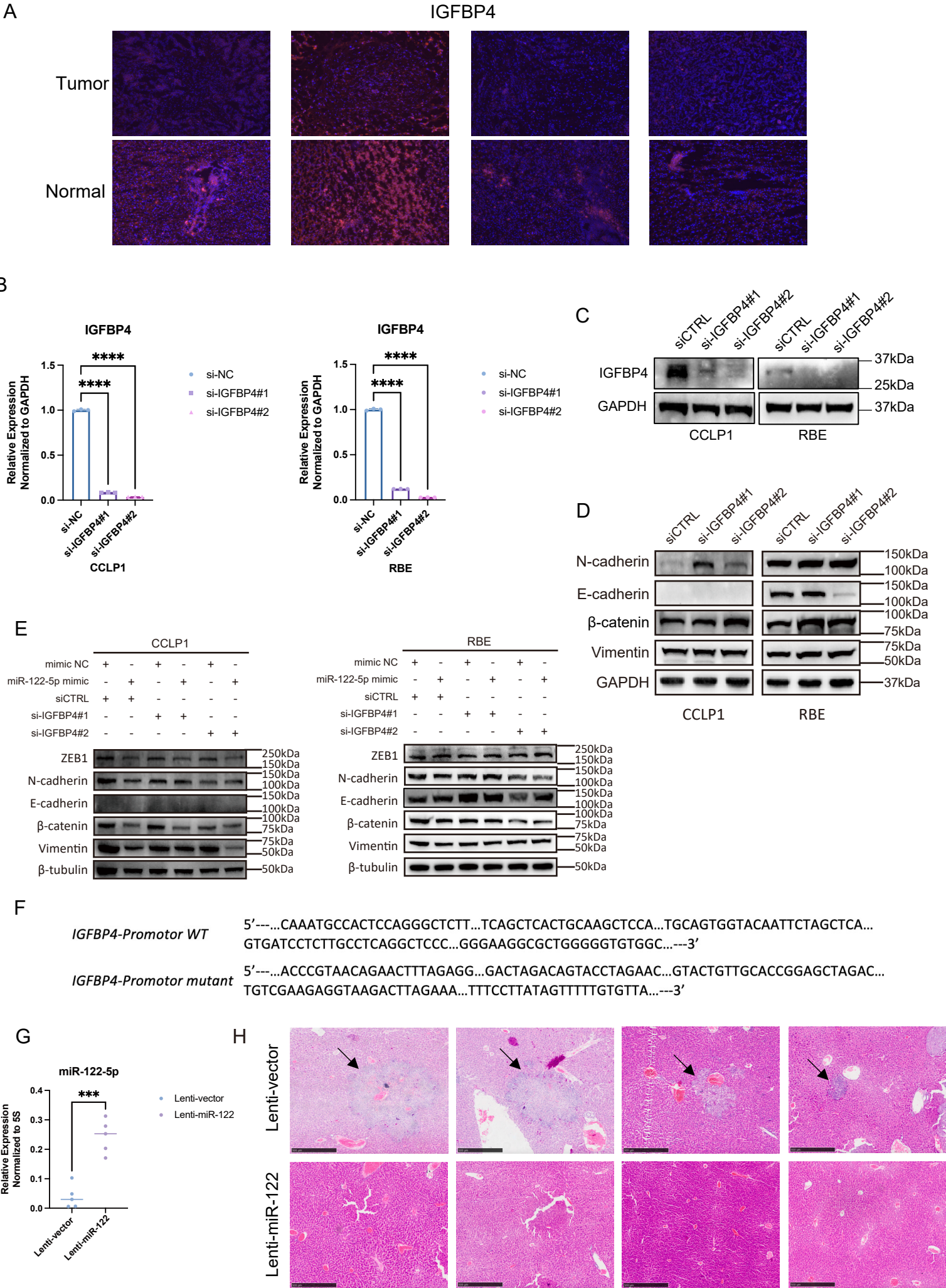

Supplement: Supplementary file 1 — Figure S1 Supplementary data for miR-122-5p is associated with invasion in intrahepatic cholangiocarcinoma and is decreased in tumor. (A) miRNA dendrogram showing the co-expression modules. (B) Relationships of consensus MEMs and clinical traits. (C) The plot shows the scale-free topology fit index (y-axis) for different soft-thresholding powers (β) (x-axis). Analysis of the mean connectivity (degree, y-axis) for various soft-thresholding powers (x-axis). (D) Module Eigenvector Clustering of 9 modules. Figure S2 Supplementary data for miR-122-5p is associated with invasion in intrahepatic cholangiocarcinoma and is decreased in tumor.(A) Volcano plot of miRNA in SRRSH ICC database. (B) Volcano plot of miRNA in TCGA CHOL database. (C) miR-122-5p is also down regulated in other types of cancer in TCGA database. (D) Differential expression of miR-122-5p in CCA samples in TCGA databases. The abscissa represents sample type and the ordinate represents gene expression. The orange box indicates tumor sample and purple box indicates normal sample (P < 0.05). (E) Differential expression of miR-122-5p on nodal metastasis status in TCGA databases. Figure S3 The efficacy of 13 hub miRNAs in diagnosing perineural invasion. (A) The efficacy of miR-122-5p in diagnosing perineural invasion. (B) The efficacy of Let-7c-3p in diagnosing perineural invasion. (C) The efficacy of Let-7c-5p in diagnosing perineural invasion. (D) The efficacy of miR-122-3p in diagnosing perineural invasion. (E) The efficacy of miR-378a-3p in diagnosing perineural invasion. (F) The efficacy of miR-378a-5p in diagnosing perineural invasion. (G) The efficacy of miR-505-3p in diagnosing perineural invasion. (H) The efficacy of miR-505-5p in diagnosing perineural invasion. (I) The efficacy of miR-574-3p in diagnosing perineural invasion. (J) The efficacy of miR-1468-5p in diagnosing perineural invasion. (K) The efficacy of miR-5589-3p in diagnosing perineural invasion. (L) The efficacy of miR-5589-5p in diagnosi [file 12072_2023_10552_MOESM1_ESM.pdf]
